# Improving Water-Based Drilling Mud Performance Using Biopolymer Gum: Integrating Experimental and Machine Learning Techniques

**DOI:** 10.3390/molecules29112512

**Published:** 2024-05-26

**Authors:** Mobeen Murtaza, Zeeshan Tariq, Muhammad Shahzad Kamal, Azeem Rana, Tawfik A. Saleh, Mohamed Mahmoud, Sulaiman A. Alarifi, Nadeem Ahmed Syed

**Affiliations:** 1 Center for Integrative Petroleum Research, King Fahd University of Petroleum & Minerals, Dhahran 31261, Saudi Arabia; mobeen@kfupm.edu.sa (M.M.); nadeem.syed@kfupm.edu.sa (N.A.S.); 2 Physical Science and Engineering Division, King Abdullah University of Science and Technology, Thuwal 23955, Saudi Arabia; zeeshan.tariq@kaust.edu.sa; 3 Department of Petroleum Engineering, King Fahd University of Petroleum & Minerals, Dhahran 31261, Saudi Arabia; mmahmoud@kfupm.edu.sa (M.M.);; 4 Department of Chemistry, School of Science, University of Management and Technology, Lahore 54770, Pakistan; 5 Chemistry Department, King Fahd University of Petroleum & Minerals, Dhahran 31261, Saudi Arabia; tawfik@kfupm.edu.sa

**Keywords:** water-based mud, swelling inhibition, fluid loss, Gum Arabic, green additive, machine learning

## Abstract

Drilling through shale formations can be expensive and time-consuming due to the instability of the wellbore. Further, there is a need to develop inhibitors that are environmentally friendly. Our study discovered a cost-effective solution to this problem using Gum Arabic (ArG). We evaluated the inhibition potential of an ArG clay swelling inhibitor and fluid loss controller in water-based mud (WBM) by conducting a linear swelling test, capillary suction timer test, and zeta potential, fluid loss, and rheology tests. Our results displayed a significant reduction in linear swelling of bentonite clay (Na-Ben) by up to 36.1% at a concentration of 1.0 wt. % ArG. The capillary suction timer (CST) showed that capillary suction time also increased with the increase in the concentration of ArG, which indicates the fluid-loss-controlling potential of ArG. Adding ArG to the drilling mud prominently decreased fluid loss by up to 50%. Further, ArG reduced the shear stresses of the base mud, showing its inhibition and friction-reducing effect. These findings suggest that ArG is a strong candidate for an alternate green swelling inhibitor and fluid loss controller in WBM. Introducing this new green additive could significantly reduce non-productive time and costs associated with wellbore instability while drilling. Further, a dynamic linear swelling model, based on machine learning (ML), was created to forecast the linear swelling capacity of clay samples treated with ArG. The ML model proposed demonstrates exceptional accuracy (R^2^ score = 0.998 on testing) in predicting the swelling properties of ArG in drilling mud.

## 1. Introduction

The prime objective of drilling muds is to control the formation damage, maintain the wellbore stability, transport the drill cuttings, and lubricate and cool the drill bit [1,2]. Clay swelling due to the porous structure of rock is of special concern for petroleum chemists. Drilling muds consist mainly of bentonite and some other property-controlling additives such as fluid loss controllers, shale swelling inhibitors, hardness removers, alkalinity controllers, and weighting material [3,4]. Water-based drilling muds (WBMs) display higher fluid loss, poor rheological features, bad swelling inhibition if not addressed. Therefore, some special property-controlling drilling mud additives such as polymers can help to attain the requirements for the drilling operation [5]. There are many additives utilized to control shale swelling during drilling operations, including inorganic salts, polymers, surfactants, ionic liquids, nanomaterials, silicates, and nanocomposites [6,7,8,9,10,11,12,13,14]. As environmental protection requirements are becoming increasingly strict at drilling sites, non-toxic and degradable biopolymers have attracted the scientific community’s attention. The swelling control, rheology, and fluid loss control of WBM could be affected by water-miscible natural gums [15].

**Figure 1:**
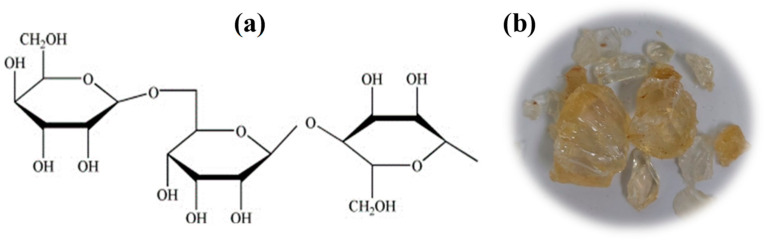
(**a**) Generic chemical structure of ArG [19]; (**b**) ArG in its raw form.

Natural gums, such as cellulose, starch, and xanthan gum, are seen as more environmentally friendly than synthetic ones and have been shown to actively improve the rheological characteristics and fluid loss control of WBM [16]. Though it has a lot of potential for research, the use of natural gums in drilling mud operations is hardly studied. The use of gums to improve the rheological characteristics and fluid loss management of drilling muds is not well documented in the literature [17]. In the current work, ArG is used as a natural biopolymer that is inexpensive, effective, and environmentally friendly to suppress clay swelling in water-based drilling mud. The ArG tree (Acacia Senegal) is the primary producer of this water-soluble polysaccharide [18]. The structure and the raw form of the ArG are shown in Figure 1a [19] and Figure 1b, respectively. ArG has been used as an emulsifying agent in the literature [20,21,22]. A thorough review of the literature proved that ArG was never used as a clay swelling inhibitor in drilling muds. We used ArG which we locally purchased at a market in Dammam, Saudi Arabia, to demonstrate its ability to suppress clay swelling. ArG can enhance the strength of the filter cake by binding to inorganic solid particles and also maintain the desired rheological properties across a broad range of temperatures [23]. The Sustainable Development Goals (SDGs) serve as a gauge for a planned process’s environmental viability [24]. Focusing on affordable and cleaner energy (SDG 7), industry, innovation, and infrastructure (SDG 9), and climate action (SDG 13), this study accomplishes numerous SDGs [25,26].

In this study, we investigated the inhibitory potential of ArG on clay swelling, a critical factor in maintaining wellbore stability during drilling operations. We performed conventional investigations, such as the capillary suction timer and linear swell tests, to evaluate the effectiveness of ArG as a clay swelling inhibitor. Additionally, we evaluated how ArG affected the rheological characteristics and fluid loss prevention potential of drilling muds. Our evaluation focused on both bentonite-based drilling mud and field mud enriched with various drilling mud additives. This investigation aimed to assess the practical applications of ArG in drilling mud formulations and its role in maintaining wellbore stability.

## 2. Results and Discussion

### 2.1. Material Characterization

**Figure 2:**
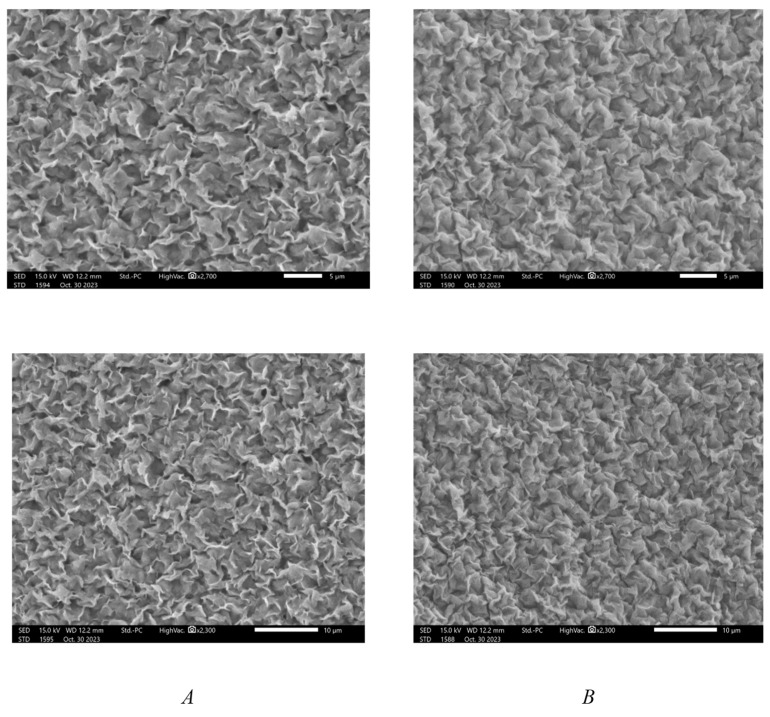
SEM images of clay without exposure to ArG (**A**) and clay after exposure to ArG (**B**).

Figure 2 shows the SEM images of the modified and unmodified WBM. Na-Ben clay might become wet and swollen when it binds to water strongly. As seen in Figure 2A, Na-Ben which had been exposed to water had an uneven surface with evident holes. However, the addition of ArG results in a smooth and compact surface with minimum pores (Figure 2B). The reduction in surface roughness indicated that Gum Arabic played a role in minimizing the irregularities and voids present on the clay surface. This phenomenon can be accredited to the adsorption of Gum Arabic onto the clay particles, leading to a more uniform and stabilized surface structure. It can be inferred from the SEM data that ArG adsorbs on Na-Ben clay; as a result, there is a prominent decrease in swelling. The protective layer formed on the surface of Na-Ben clay prevents its interaction with water.

**Figure 3:**
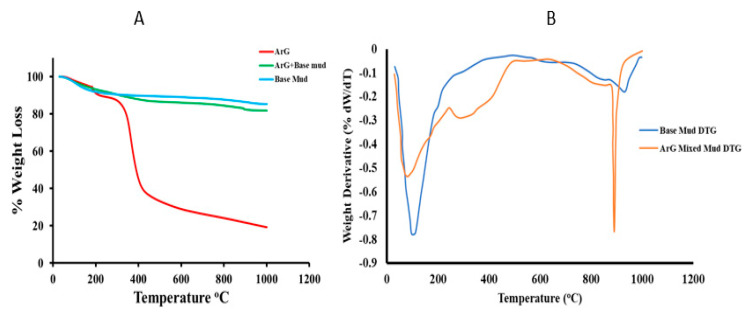
Thermogravimetric analysis of ArG, base mud, and 1.0 wt. % ArG mixed mud (**A**) and derivative of the TGA curve (DTG) for base and ArG mixed muds (**B**).

Information regarding the material’s thermal stability could be obtained from the TGA. The behavior of the base mud, ArG, and ArG combined mud is shown in Figure 3. A further mass derivate was calculated for base and ArG mixed muds, as shown in Figure 3B. According to the TGA (Figure 3A,B), weight loss of around 13% results from dehydration or desorption of remaining moisture at temperatures between 30 °C and 300 °C. As shown in the literature, at this point, the hydrogen-bonded water underwent desorption from polysaccharides [27]. For ArG, between 325 °C and 425 °C, the first significant weight loss of 45–50% takes place [28]. The significant decrease in ArG weight suggests that natural polysaccharides break down. As a result of the breakdown, H_2_O, CO, and CH_4_ are generated [29,30]. At 1000 °C, the final residue included 23 wt. % ArG. Nevertheless, the base mud showed superior stability; the mass loss was 15% up to 1000 °C, with water desorption accounting for the majority of the mass loss. There was a noticeable behavioral shift when ArG and base mud were mixed. Up to 290 °C, the mixture’s TGA showed enhanced thermal integrity. Above it, there was more loss than base mud due to thermal degradation of the adsorbed ArG on the bentonite. Consequently, 81% residue was obtained from the ArG mixed base mud mixture at 1000 °C.

**Figure 4:**
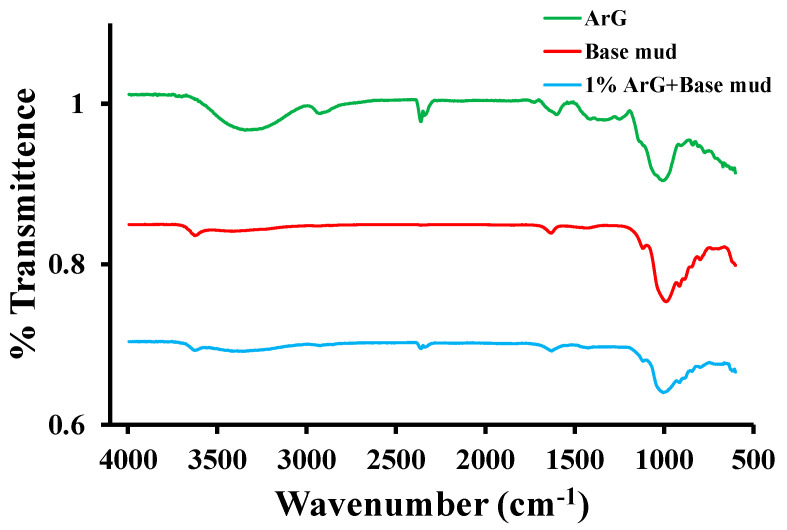
FTIR spectra of ArG, base mud, and 1.0 wt. % ArG+base mud.

The FTIR spectra of ArG, base mud, and 1.0 wt. % ArG+base mud are displayed in Figure 4. A distinctive broad absorption band at 3334–3390 cm^−1^ was observed, indicating the presence of a hydrogen-bonded OH group [31]. The broad absorbance band of the O-H group covers the amino group’s band, present in the range of 3400–3500 cm^−1^ [27]. The bands at 2906 cm^−1^ indicate the C-H stretching of the sugars galactose, arabinose, rhamnose, alkane, and aldehyde. Additionally, the polymers exhibited the characteristic band of the C=C stretch, the amide NH bend, the NO_2_ from aliphatic and aromatic galactoproteins, and amino acids at 1592 cm^−1^. The peaks observed at 1173 cm^−1^ represented C-O, C-C, and C-O-C stretching, as well as and C-O-H and C-H bending modes of the polymer backbone, as reported previously [32,33,34]. A distinct band at approximately 1007 cm^−1^ represents the alkene C-H bend from polysaccharides [35]. The base mud demonstrated –OH stretching at 3636 cm^−1^ and 3352 cm^−1^, which confirms the hydrophilic nature and capacity of base mud to absorb water [36]. The angular vibration of O–H of water due to hydration shows an absorption band at 1634 cm^−1^. The mixing of base mud with 1.0 wt. % ArG causes adsorption of the materials that results in the prominent change in the surface features of the individual materials. A decrease in the absorption band at 3552 and 1630 cm^−1^ was observed related to the –OH group of hydration water. There is also a prominent alteration in the Si–O band observed that is due to the strong binding of ArG with the base mud. Interestingly, the absorption peak associated with the Al–O bending in base mud shifts from 516 cm^−1^ to 470 cm^−1^, which can be ascribed to the formation of hydrogen bonding between hydroxyl on the ArG’s pyranose ring and silanol on the clay surface [37,38]. Results from the FTIR data verify that adding ArG to the base mud enhances ArG adsorption because of hydrogen bonding.

### 2.2. Swelling Inhibition

**Figure 5:**
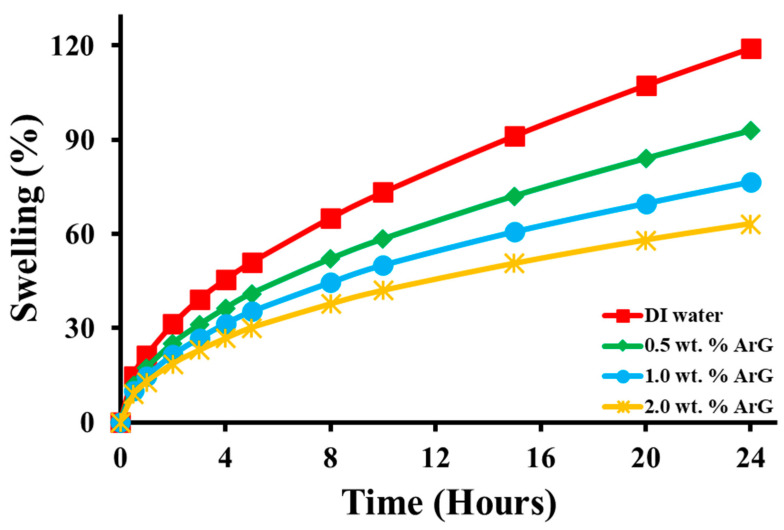
The linear swelling evaluation results of base mud, 0.5 wt. % ArG+base mud, 1.0 wt. % ArG+base mud, 1.0 wt. % ArG+base mud, and 2.0 wt. % ArG+base mud.

Figure 5 illustrates the linear expansion of Na-Ben under various inhibition mediums. The clay exhibited the highest swelling, reaching 119.4%, when exposed to deionized water. However, the introduction of 0.5 wt. % ArG decreased the swelling by 26.1%. With increasing ArG concentrations to 1.0 wt. % and 2.0 wt. % the linear expansion reduced to 76.6% and 63.29%, respectively. Notably, the addition of 2.0 wt. % ArG led to the most significant reduction in swelling for bentonite pallets, with a decrease of 63.3%. This reduction indicates the robust hydrogen bonding of ArG with the clay, confirming ArG’s inhibitory performance. Further, the swelling inhibition was directly proportional to the concentration of ArG.

**Figure 6:**
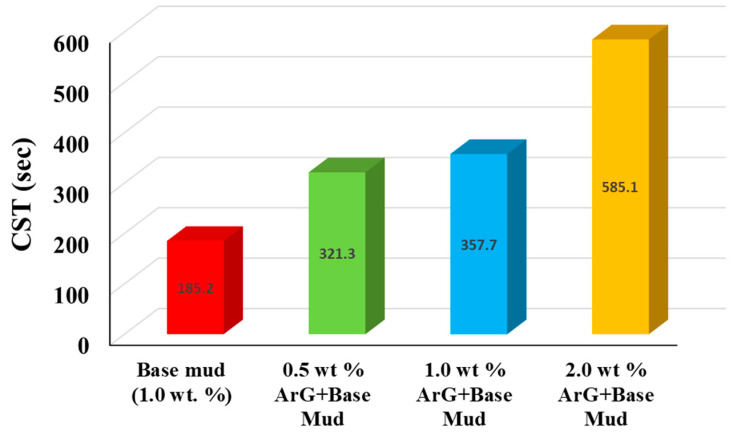
Base mud, 0.5 wt. % ArG+base mud, 1.0 wt. % ArG+base mud, 1.0 wt. % ArG+base mud, and 2.0 wt. % ArG+base mud CST results.

By monitoring the passage of filtrate across the filter media, the CST test determines the inhibition potential [7]. Figure 6 displays the CST results for both modified and unmodified mud. Because Na-Ben binds to water more extensively in the base mud, it showed the lowest CST time. On the other hand, a longer CST time was noted following the addition of ArG to the drilling mud. When the amount of natural gum in the base fluid increases, so does the CST time. It illustrates how adding ArG lowers fluid loss and makes the filtrate move from one set electrode to another more slowly than the base fluid; 185.2 s was the time for the base drilling mud. The CST time was raised to 321.3 s, 357.7 s, and 585.1 s, respectively, for 0.5, 1, and 2 wt. % ArG solutions. To stop water from passing through the filter cake and extend the travel time, the gum forms a coating on it.

The zeta potential measurement of the base mud (Table 1) demonstrates the average surface charge of the clay. The base mud (0.5 wt. % bentonite in water) displayed a zeta potential value of −35.42 mV. However, the addition of 0.5 wt. %, 1.0 wt. %, and 2.0 wt. % of ArG to the base mud showed zeta potential values of −30.74 mV, −27.29 mV, and −22.72, respectively. The sign of the zeta potential of the base mud is determined by the predominant functional groups present on the surface of the particles in the mud. Zeta potential arises due to the presence of charged groups or ions on the clay surface, which interact with the surrounding fluid. The clay surface is predominantly negatively charged, so the zeta potential will be negative. The decrease in the negative zeta potential values of the base mud upon the addition of ArG can indeed be attributed to the adsorption of ArG onto the clay particles. ArG is made up of a complicated polysaccharide chain, arabinose, and galactose units, together with side chains containing various functional groups such as carboxyl and hydroxyl. The latter mentioned are, in most cases, charged functional groups that have the potential to bear influence on the overall charge of the molecule and, in turn, the zeta potential. Generally, in its aqueous solution, ArG has negative zeta potential values, mainly at or below neutral pH. The negative charges come from carboxylic groups and other ionizable groups present on its molecular structure. These groups may become ionized and therefore confer a general negative charge to the Gum Arabic molecule.

The adsorption of ArG onto the clay particles effectively neutralizes some of the negative charges on the particle surfaces, resulting in a reduction in the magnitude of the zeta potential. This phenomenon occurs because the negatively charged functional groups of ArG compete with the clay particles for the positively charged sites on the surface, effectively reducing the net surface charge of the particles. As a result, the zeta potential becomes less negative with increasing concentrations of ArG in the mud. This decrease in zeta potential indicates a decrease in the overall surface charge density of the clay particles due to the adsorption of ArG, which is consistent with the behavior of negatively charged polymers like ArG in aqueous solutions.

### 2.3. Rheology and Fluid Loss Tests

Studying the rheological properties can provide crucial information about the clay swelling inhibitor. Plastic viscosity (PV) is the measure of the internal resistance of the particles in drilling mud during flow. The solid components present in drilling mud significantly affect PV. Drilling muds with lower PV values are preferred as they enable faster drilling. As shown in Table 2, the addition of 1.0 wt. % ArG results in a slight decrease in the PV of the drilling mud, showing a friction reduction effect. In colloidal suspensions of WBM, the yield point (YP) is affected by the interparticle forces as well as the fluid friction. A greater YP in the drilling fluids may cause partial flocculation and blockage in the drilling system. On the other hand, a drop in YP may signify a decline in the drilling mud’s flocculation [39]. ArG-modified drilling mud had a lower YP than unmodified base mud. Gel strength can be used to evaluate the attractive forces present in water-based muds in the absence of flow. The 10 s gel and 10 min gel had minor alterations upon the introduction of ArG because of a reduction in attractive forces. Measuring the ratio of YP/PV is essential for determining the degree of shear thinning in drilling muds. This measurement is significant because it helps in assessing the mud’s ability to clean the well and the required pumping pressure [40]. Table 2 shows that the addition of ArG has no effect on the ratio of YP/PV. As a result, it can be inferred that the incorporation of ArG into WBM substantially enhances its ability to transport cuttings, making it a more appropriate choice for drilling operations. Further ArG additions did not bring much change in the drilling mud properties. It showed compatibility with other additives.

**Figure 7:**
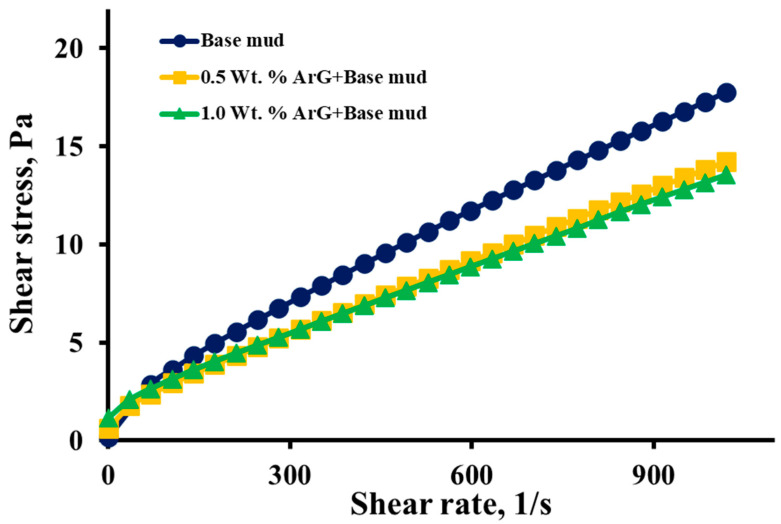
Base mud, 0.5 wt. % ArG+base mud, and 1.0 wt. % ArG+base mud shear rate–shear stress plots.

The shear stress–shear rate curves of the modified drilling mud samples and the bentonite base are plotted in Figure 7. It is observed from the graph that the stress values at a given shear rate were decreased after the incorporation of ArG when compared to the control. The base sample resulted in high shear stresses in the high shear rate range. With the increase in ArG concentration, the shear stresses were reduced, and its shear stress–shear rate curve was lowered.

The study of rheological features revealed that the addition of ArG to the base mud did not cause any prominent change. The addition of ArG to the base sample slightly increased its yield point. Such a meager change does not affect the performance of the drilling mud.

**Figure 8:**
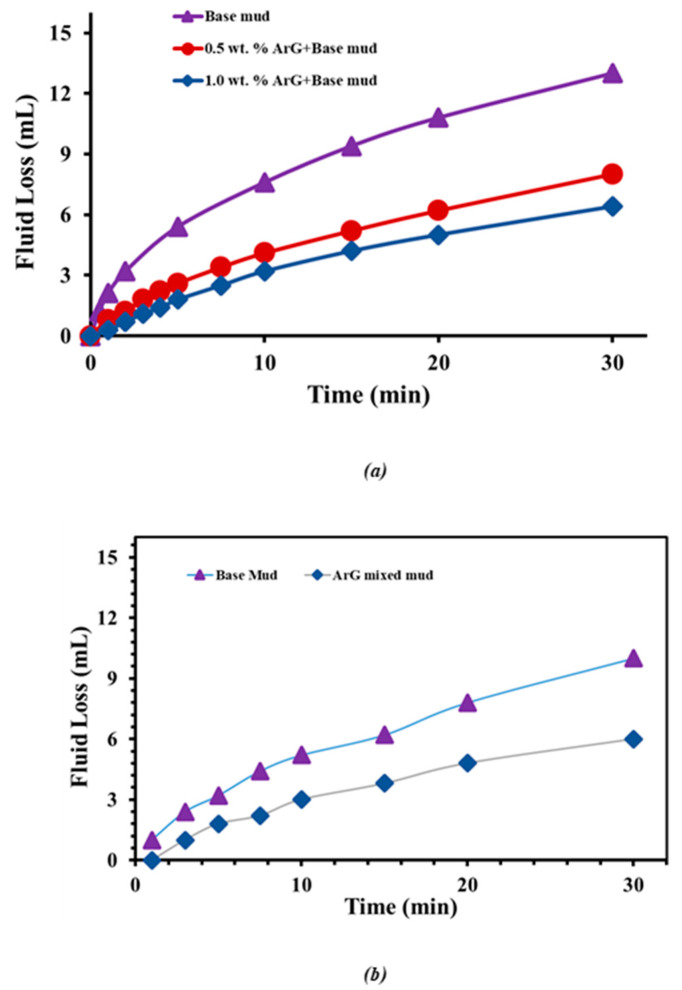
Fluid loss test of (**a**) bentonite mud, 0.5 wt. % ArG+bentonite mud, and 1.0 wt. % ArG+bentonite mud. (**b**) Base mud and base mud modified with 1.0 wt. % ArG.

The purpose of the fluid loss test was to assess ArG’s potential as a fluid loss controller and how it might affect the performance of the drilling mud. The outcomes of the modified and base drilling muds are shown in Figure 8. After 30 min, the bentonite-based drilling mud (Figure 8a) had a significant fluid loss of 13 mL. The fluid loss was significantly decreased by adding ArG to the bentonite drilling mud. The results showed that the control of fluid loss depends on ArG content. When 0.5 wt. % ArG and 1.0 wt. % ArG is added, the fluid loss decreases from 13.0 mL to 8.0 mL and 6.4 mL correspondingly. A similar performance of ArG was observed in drilling mud mixed with different additives. The base mud already contains various additives (Table 2); therefore, unmodified drilling mud displayed fluid loss of 10 mL after 30 min (Figure 8b). The base drilling mud’s ability to limit fluid loss is significantly impacted by the addition of 1.0 weight percent ArG. A 40% decrease in fluid loss was observed compared to the unmodified base fluid (Table 2). The decrease in fluid loss indicates the effectiveness of ArG in controlling and inhibiting fluid loss. This is because ArG forms a protective coating on the surface of the clay, reducing the volume of filtrate that may enter the formation and causing the least formation damage. The shale has less water available for hydration and dispersion due to the reduced fluid loss into the shale formation.

### 2.4. Inhibition Mechanism

**Figure 9:**
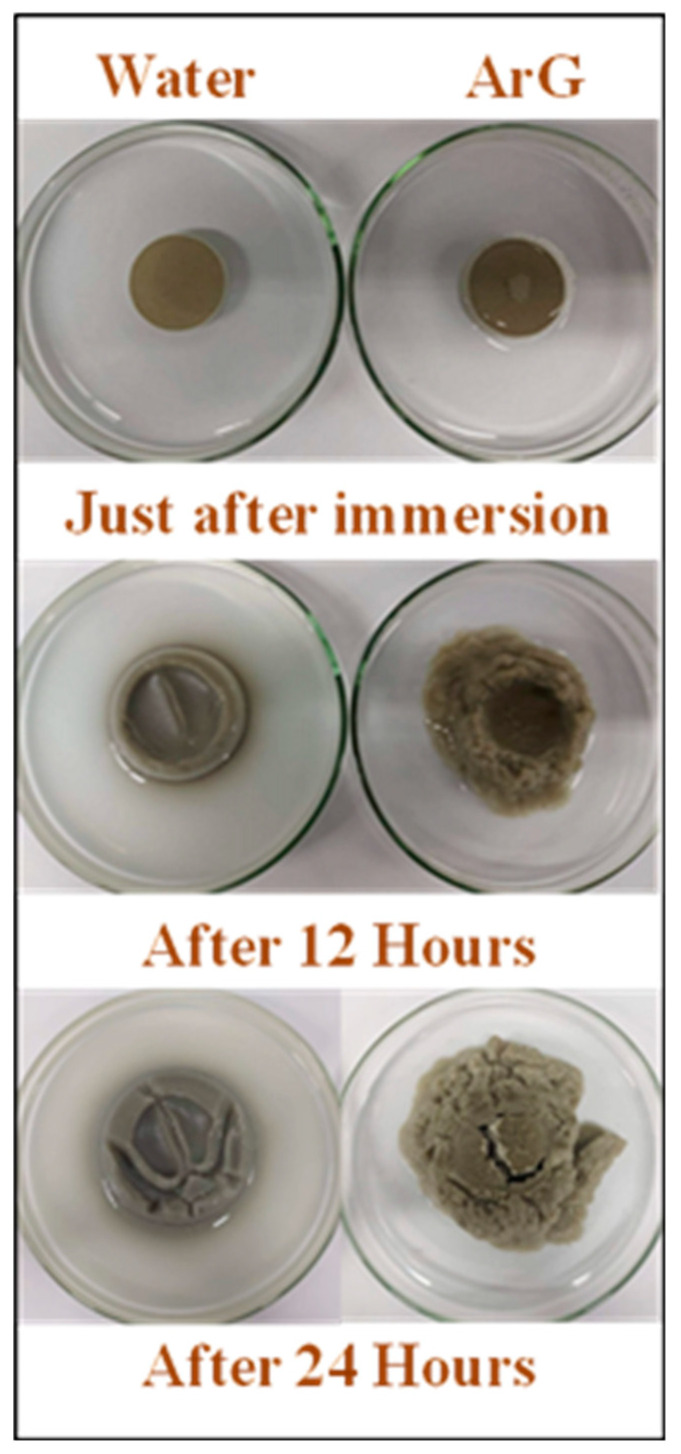
The wetting test of clay pellet in water and ArG.

Strong binding of the ArG to the clay surface was demonstrated by the thorough characterization using advanced techniques and evaluation of rheological and swelling inhibition properties. In order to collect further evidence, the time-based wetting test for the Na-Ben pellet was carried out (Figure 9). Clay pellets were prepared and placed in separate containers with water and the aqueous solution of ArG. The container was closely monitored, and top-view images were captured at specific intervals. Initial images were taken after the pellets were immersed in the respective solutions to establish a reference size and shape for comparison. The Na-Ben pellets exhibited swelling from the edges, with less prominent swelling observed in the ArG aqueous solution. After 12 h, the pellet in water showed significant lateral swelling and an increase in height. Moreover, the bentonite particles separated from the bulk, and a dispersion of Na-Ben was formed. In contrast, the Na-Ben pellet in the ArG solution exhibited minimal lateral or height swelling. Although some pellet degradation occurred, there were no soluble particles or clay dispersion. This suggests that ArG effectively binds to the clay surface via adsorption, reducing clay disintegration, swelling, and particle dispersion. After 24 h, the Na-Ben pellet in water exhibited a significant increase in size and height, while the Na-Ben pellet in the ArG solution displayed minimal swelling and clay particle dispersion. The appearance of minimal clay particle dispersion in the presence of ArG can be accredited to the partial extinction of Na-Ben charge due to binding with the ArG that helps to overcome the mutual repulsion of clay particles.

**Figure 10:**
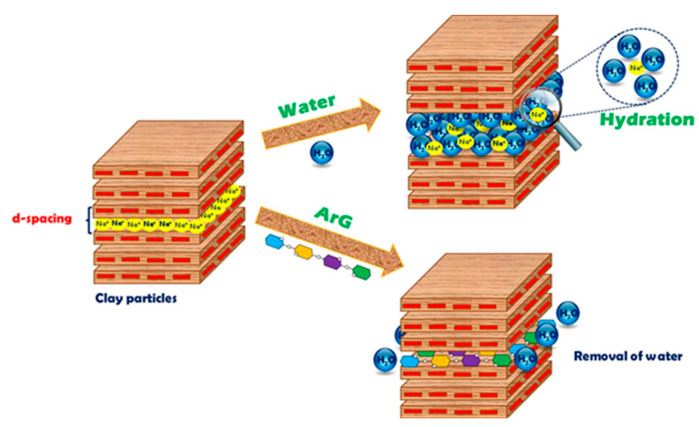
Schematic representation of the inhibition mechanism by ArG.

Extensive characterization, rheological investigation, and evaluations of swelling inhibition showed that ArG is capable of protecting Na-Ben from water. Developing a tenable inhibitory mechanism for ArG that is consistent with the analysis’s findings is the main goal of this section. The study results indicated that the hydrogen bonding of ArG with clay is crucial in inhibiting clay swelling. The active constituents of ArG facilitate intercalating and adsorbing onto the clay surface via physicochemical interactions through hydrogen bonding [41]. Consequently, the protective layer of ArG makes the hydrophilic clay surface more water-resistant (Figure 10).

Table 3 compares the prominent features of ArG with the other shale inhibitors reported in the literature. The swelling rate, rheological features, and fluid loss control of the modified cellulose and biopolymers were studied. An overall improvement in linear swelling control, rheological features, and fluid loss control was observed.

## 3. Machine Learning Modeling

In this study, a total of 493 data points were recorded during the laboratory experiments. The dataset underwent thorough statistical analysis [46,47]. This analysis is listed in Table 4.

**Figure 11:**
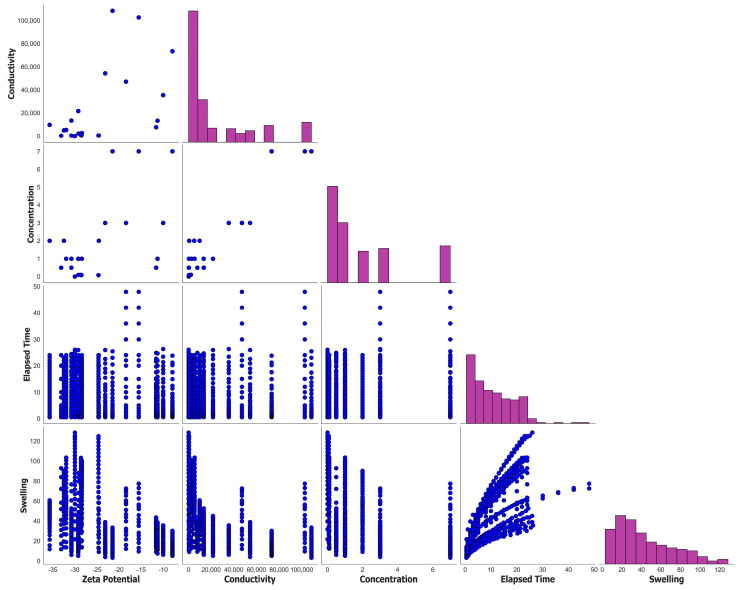
Pair plot of the total dataset.

Figure 11 displays the pair plot of the entire dataset used for the predictive modeling using machine learning (ML) techniques. The use of a pair plot can provide an initial overview of the dataset.

**Figure 12:**
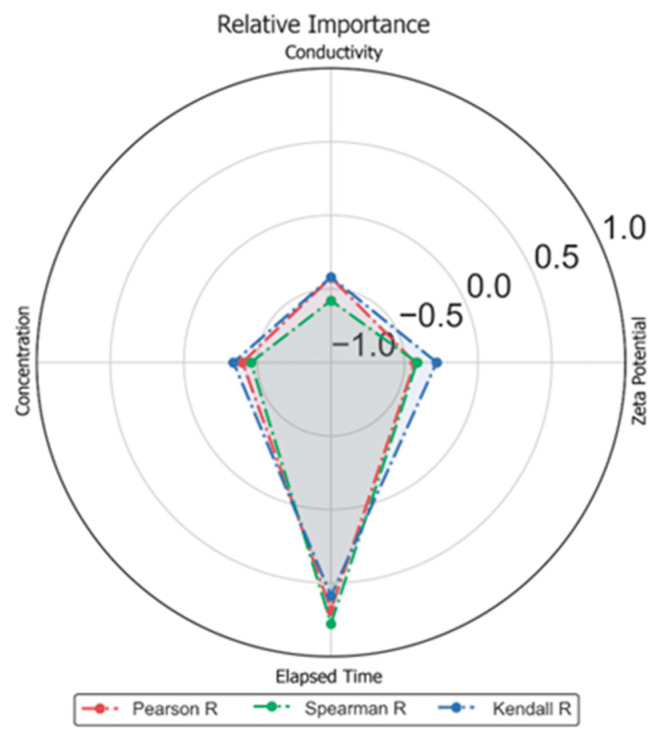
Strength of input parameters against dynamic linear swelling.

Figure 12 shows the impact of input features with the linear swelling in terms of radar plot in terms of Pearson, Spearman, and Kendall’s correlation coefficient criteria, defined as Equations (1)–(3).

(1) $$\rho_{pearson}=\frac{n_{samples}\sum xy-\left(\sum x\right)\left(\sum y\right)}{\sqrt{n_{samples}\left(\sum x^{2}\right)-{\left(\sum y\right)}^{2}}\sqrt{n_{samples}\left(\sum x^{2}\right)-{\left(\sum y\right)}^{2}}}$$

(2) $$\rho_{spearman}=\rho_{pearson}\frac{cov\left(x,y\right)}{\gamma_{x}\gamma_{y}}$$

(3) τkendall=nc−ndnsamplesk−12

where x and y are the input variables, $n_{samples}$ is the total number of samples, $cov\left(x,y\right)$ is the covariance of the rank variables, $\gamma_{x}\gamma_{y}$ are the standard deviations of the rank variables, $n_{c}$ is the number of concordant pairs, and $n_{d}$ is the value of the number of discordant pairs.

**Figure 13:**
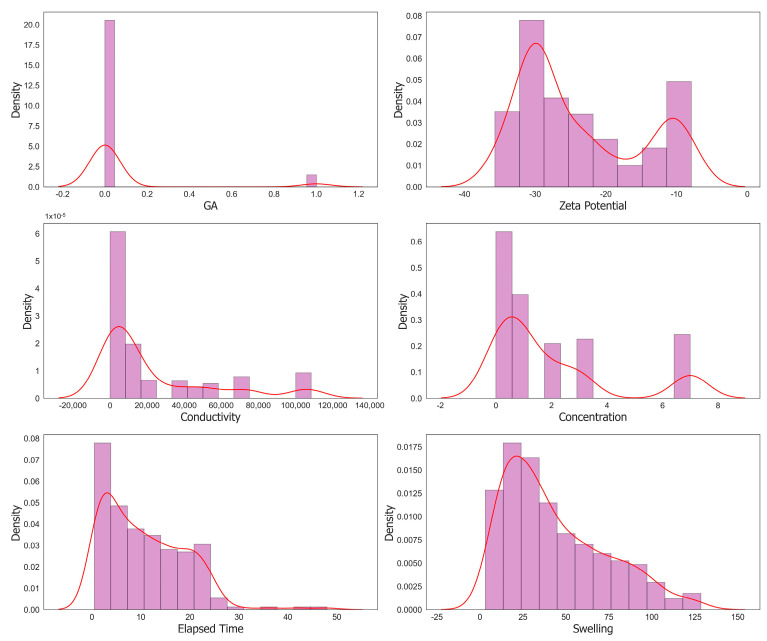
Histograms of input and output parameters used for machine learning model training.

**Figure 14:**
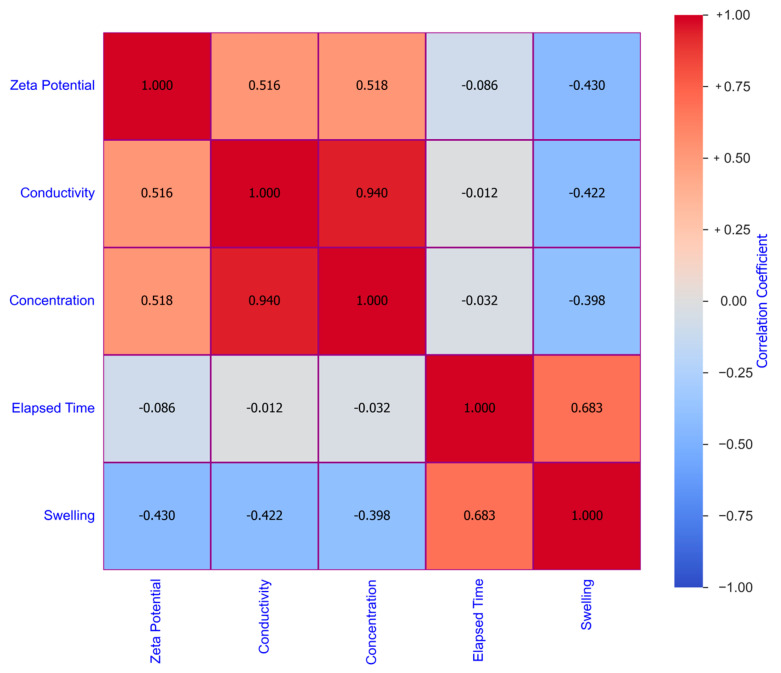
Display of Pearson correlation coefficient among the variables in a total dataset using a heatmap.

Figure 13 shows the entire distribution of input parameters using histograms. To assess the collinearity among input parameters, a heatmap using Pearson’s correlation method was generated. Here, collinearity refers to a high degree of correlation or linear dependency between two or more input parameters. Figure 14 displays the heatmap of all the parameters.

**Figure 15:**
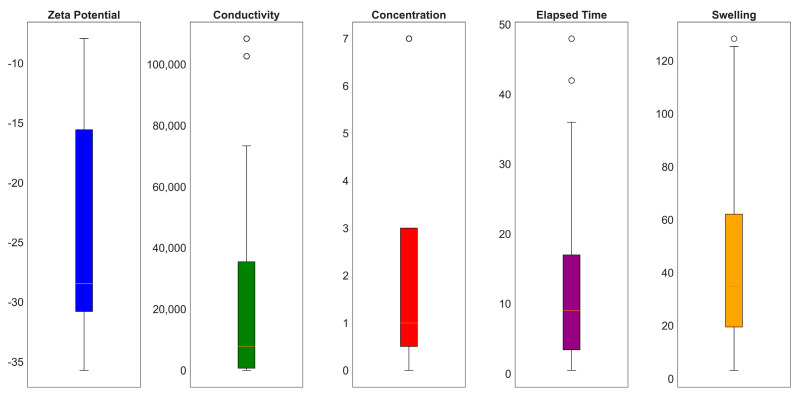
Box plot of the input parameters.

In Figure 15, the non-categorical variables are represented by violin plots. These plots present the mean and interquartile ranges (IQRs). The IQR was computed by subtracting the 75th percentile (Q3) from the 25th percentile (Q1), denoted as IQR = Q3 − Q1.

**Figure 16:**
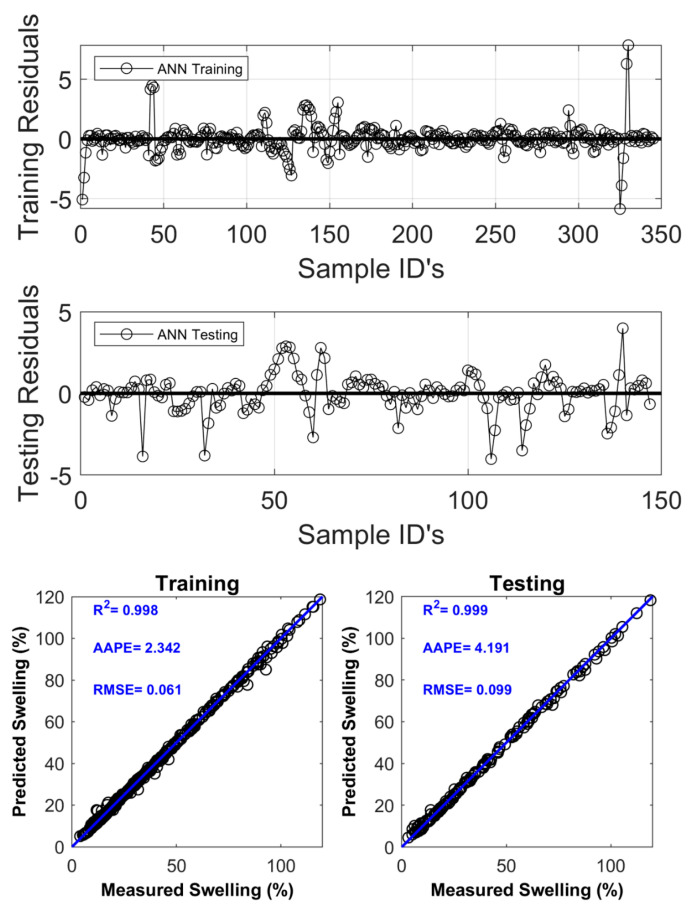
Training and testing prediction of linear swelling with ANN.

This study utilized feedforward neural networks (FNNs) to model dynamic linear swelling. In FNNs, a message moves in the forward direction, from the input layer to the output layer, without any loops or feedback connections. The hyperparameters of the trained FNN model are given in Table 5. The FNN model inputs properties such as zeta potential, conductivity, and swelling time. Figure 16 illustrates the output of the FNN model’s predictions for linear swelling in both the training and testing stages. In the training phase, the FNN model attained an AAPE of 2.342%, R^2^ of 0.998, and RMSE of 0.061. In the testing phase, the FNN model produced an AAPE of 4.191%, R2 of 0.999, and RMSE of 0.099.

**Figure 17:**
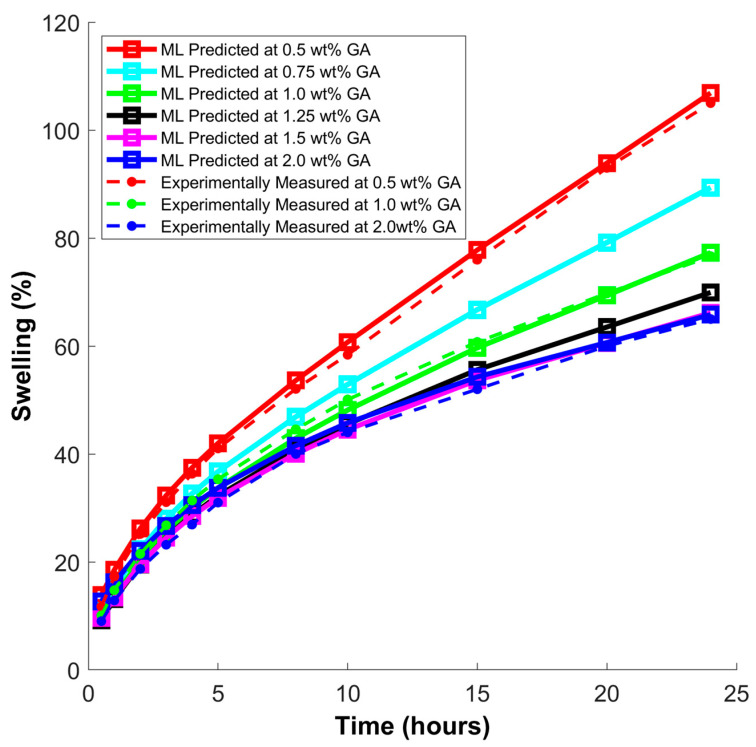
Swelling behavior over a period of 24 h.

Swelling curves were generated using the trained FNN model to analyze the linear swelling trend of shale samples treated with various concentrations of green polymer in WBDFs, as shown in Figure 17. To achieve this, a new dataset was generated with only one input parameter being changed in each dataset. The swelling response was forecasted and graphed over time for various concentrations, including, 0.5 wt. %, 0.75 wt. %, 1.0 wt. %, 1.25 wt. %, 1.5 wt. %, and 2.0 wt. %. The predictions of the FNN model matched the experimentally measured findings recorded at 0.5 wt. %, 1.0 wt. %, and 2.0 wt. %, indicating that as the polymer concentration increases, the rate and ultimate linear swelling in shales decreases.

## 4. Materials and Procedures

### 4.1. Materials

**Figure 18:**
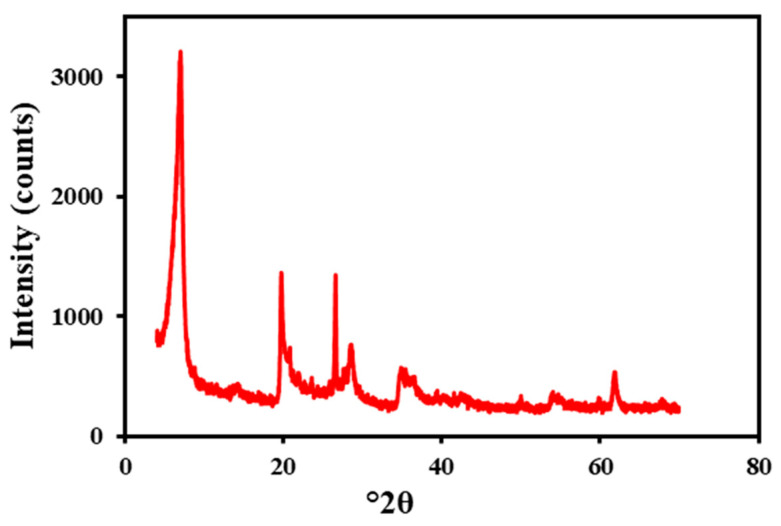
XRD of sodium bentonite.

ArG was purchased from local markets and used as an inhibitor for clay swelling. The pH was adjusted using NaOH, which was provided by Sigma Aldrich. Figure 18 displays the XRD of the bentonite. Na-Bentonite (Na-Ben) was utilized to investigate the modified WBM’s inhibitive potential. Bentonite is composed of various mineral components, with the following approximate composition by % mass: 54% montmorillonite, 15% quartz, 12% illite, 9% K-feldspar, 7% muscovite, 2% gypsum, and 1% cristobalite. All the solutions were mixed using deionized water.

The gum’s attributes are detailed in Table 6. Galactose, arabinose, rhamnose, and glucuronic acid are among the several saccharides that it contains [48].

### 4.2. Preparation of Drilling Mud

The preparation and testing of modified WBMs were conducted according to the experimental protocols described in our earlier work [49]. In this work, two different drilling mud formulations were tested, one bentonite-based drilling mud and one field drilling mud with different additives.

To formulate the bentonite drilling mud (BM), a mixture of 6 wt. % Na-Ben in water was made. For the ArG-modified drilling muds (ArGM), ArG (ArG) at concentrations of 0.5 wt. % and 1.0 wt. % was added to the 6 wt. % Na-Ben solution. To ensure uniformity, vigorous stirring was carried out for 30 min. To keep the mixture at a pH of 9, NaOH was also added.

In the case of field drilling muds enriched with various additives, modified and unmodified WBM were prepared. The composition included 10.0 g of bentonite, 0.5 g of XC polymer, 0.4 g of starch, 50.0 g of barite, 0.2 g of NaOH (to maintain a pH of 10), and 3.5 g of ArG, as specified in Table 7. The components of the field drilling mud were thoroughly mixed for different time intervals to achieve a homogeneous mixture.

### 4.3. Material Characterization Techniques

A high-resolution scanning electron microscope (SEM) Helios FIBSEM was used to evaluate the size, shape, and surface characteristics of the sample particles. For the characterization of vibrational features of functional groups present in the shale sample, a Fourier Transform Infrared (FTIR) spectrometer from PerkinElmer was used. The stability of the materials under study was analyzed by thermogravimetric analysis (TGA). A thermogravimetric study (TGA 8000-PerkinElmer, Waltham, MA, USA) was carried out up to 700 °C at an increased rate of 10 °C/min with continuous nitrogen flow (40 mL/min).

#### 4.3.1. Swelling Inhibition Tests

To study swelling inhibition, three different tests were conducted: a linear swell test (LST), a capillary suction timer (CST), and a zeta potential test. By utilizing a dynamic linear swell analyzer from OFITE, a linear swell test was carried out to examine the sodium bentonite pellet’s expansion linearly in the presence of an aqueous fluid of ArG [49]. Three different concentrations of ArG were studied, including 0.5, 1, and 2 wt. %. The test was conducted in room conditions. The swelling of clay in deionized water was taken as a reference. In this experiment, a bentonite pellet was made using 12 g of bentonite that had been hydraulically compressed at 6000 psi and then exposed to non-inhibitive and inhibitive fluids. For 24 h, linear expansion was observed. In the end, a comparison between inhibitory and non-inhibitory aqueous fluid performance was made.

The practical inhibitory potential of the drilling mud was evaluated by the CST test. In the current work, the OFITE CST instrument was used to explore the inhibitory potential of ArG. This test calculates the amount of time it takes for a filtrate to pass through filter media and move from one set of electrodes to another. Several drilling mud compositions with varying ArG concentrations (0.5, 1, and 2 wt. %) were employed to conduct the CST test. Bentonite was mixed with deionized water to 1.0 wt. % to create the base fluid. The CST apparatus cylinder was then filled with 5.0 mL of drilling mud, with the filter media at the bottom.

Zeta potential measurement was employed to assess the surface charge of the clay in DI water and ArG solutions. Four samples were prepared for the zeta potential measurements; the first sample contained 1 g of Na-Ben clay mixed in 100 mL of deionized water, while the second sample contained 1 g of Na-Ben mixed in 100 mL of a 0.5 wt. % ArG mixed solution. The third sample was made by mixing 1 g of bentonite with a 1 wt. % solution of ArG. The fourth sample was made by mixing 1 g of bentonite with a 2 wt. % solution of ArG. Using a magnetic stirrer, the Na-Ben dispersions were stirred for 24 h to achieve full hydration of the bentonite. At 25 °C, the Litesizer 500 was used to determine the zeta potential of the prepared samples.

#### 4.3.2. Rheology and Fluid Loss Tests

The rheology of drilling muds was investigated in room conditions by following the testing procedures of the American Petroleum Institute (API-13B) [50]. The TA Hybrid Discovery rheometer was used to measure the relevant shear stresses as the shear rates were changed. Furthermore, plastic viscosity (PV), yield point (YP), and gel strength were evaluated by applying 600 rpm and 300 rpm dial readings of a viscometer in the following formulas (Table 8), as per the standard procedure for evaluation.

The low-pressure, low-temperature (LPLT) API filter press (FANN series 300) was used to perform the fluid loss test. We have published a complete procedure in our recent publication [7]. The fluid loss test took place at 100 psi of pressure and 25 °C temperature.

## 5. Conclusions

ArG is a natural biopolymer derived from the Acacia Senegal tree and is an eco-friendly, cost-effective, and plant-based additive. This study focused on demonstrating the swelling inhibition capacity of ArG and its impact on the fluid loss and rheology of drilling mud. For inhibition investigation, three different concentrations of ArG were investigated, including 0.5, 1, and 2 wt. %. The following conclusions have been made from the experimental investigations:

- The linear swelling test results revealed that the effectiveness of ArG in inhibiting swelling depended on its concentration.
- The CST results showed that ArG prevented fluid loss in a highly concentration-dependent manner.
- The zeta potential results showed that intercalation and adsorption of the ArG on the clay changed the average surface charge of the clay.
- There was no significant alteration in the rheological behavior of the base mud upon the addition of ArG. This indicated that ArG integration did not adversely affect the mud’s flow and viscosity properties.
- When ArG was introduced into the base mud, a significant improvement in fluid loss control was observed. The addition of 0.5 wt. % ArG reduced the fluid loss from 13.0 mL to 8.0 mL, and 1.0 wt. % ArG further decreased it to 6.4 mL.
- ArG is an environmentally friendly material which can be added to drilling muds to control shale swelling and fluid loss to enhance the biodegradability of the drilling fluids.
- A developed FNN (feedforward neural network) model exhibited exceptional accuracy in predicting the swelling behavior of shales treated with ArG. This finding suggests that the FNN model can be a valuable tool for accurately forecasting the impact of ArG on clay swelling, aiding in the formulation of mud systems for various industrial applications.

## Figures and Tables

**Table 1:** Zeta potential of drilling mud.

Formulations	Zeta Potential (mV)
Base mud (0.5 wt. %)	−35.42
0.5 wt. % ArG	−30.74
1.0 wt. % ArG	−27.29
2.0 wt. % ArG	−22.72

**Table 2:** Rheological properties of field drilling mud at 75oF and 14.7 psi.

Mud Formulation	PV (cp)	YP (lb/ft^2^)	YP/PV (Pa/mPA.s)	Gel_10s_ (lb/ft^2^)	Gel_10min_ (lb/ft^2^)	Fluid Loss (mL)
Unmodified Base Mud	8.4	7.5	0.9	2.6	3.7	10.0
ArG-Modified Base Mud	7.7	6.9	0.9	2.1	3.2	6.0

**Table 3:** Comparison of key characteristics of ArG with reported shale inhibitors.

Compounds	Swelling Rate Test	Rheological Features				Fluid Loss (mL)	Refs
		PV (cp)	YP (lb/ft^2^)	Gel_10s_ (lb/ft^2^)	Gel_10min_ (lb/ft^2^)		
ArG	63% reduction in swelling	7.7	6.9	2.1	3.2	6.0	This work
Styrene-modified cellulose	56% reduction in swelling	10.3	7.0	5.2	7.6	-	[42]
Poly-L-arginine	53% reduction in swelling	36	12	2.5	4.0	4.2	[43]
Gelatin	69% reduction in swelling	-	-	-	-	-	[44]
Catechol-Chitosan Biopolymer	32% reduction in swelling	10	-	-	-	80	[45]

**Table 4:** Statistics of the input parameters.

Statistical Parameters	Mean	SD	Variance	Kurtosis	Skewness	Range	Minimum	Maximum
DW	0.0549	0.2280	0.0520	13.4285	3.9210	1.0000	0.0000	1.0000
GA	0.0671	0.2504	0.0627	10.0955	3.4720	1.0000	0.0000	1.0000
Zeta Potential	−23.7771	8.7073	75.8178	−1.0824	0.6115	27.7819	−35.7088	−7.9269
Conductivity	2.34 × 10^4^	3.17 × 10^4^	1.00 × 10^9^	1.17 × 10^0^	1.53 × 10^0^	1.08 × 10^5^	1.20 × 10^0^	1.08 × 10^5^
Concentration	1.9557	2.2591	5.1036	0.7208	1.4164	7.0000	0.0000	7.0000
Elapsed Time	10.6557	8.2912	68.7443	1.1906	0.9229	47.5297	0.4703	48.0000
Swelling	43.3333	29.3014	858.5740	−0.1684	0.8392	125.3740	3.1160	128.4900

**Table 5:** Hyperparameters of the trained FNN model.

Hyperparameter	Typical Range	Optimum Value (Approximate)
Number of Hidden Layers	1 to 3	1
Number of Neurons per Layer	30	25
Activation Function	ReLU, Leaky ReLU, or PReLU	ReLU
Learning Rate	0.001 to 0.01	0.001
Epochs	100 to 1000	100
Optimizer	Adam, RMSprop, etc.	Adam
Dropout Rate	0.0 to 0.3	Requires tuning
Learning Rate Schedule	Learning rate decay, etc.	Learning rate schedule
Loss Function	Mean Squared Error (MSE)	MSE

**Table 6:** Characteristics of ArG [48].

Contents and Properties	Acacia Senegal
% Galactose	38.9
% Arabinose	25.7
% Rhamnose	9.5
% Glucuronic acid	21.5
4-O-methyl glucuronic acid	1.5
% Nitrogen	0.36
Average molecular mass	640,000
Density at 1.0 wt. % in g/cm^3^	1.0016
Viscosity at 1.0 wt. % in mpa.S	2.084
pH at 1.0 wt. %	5.52

**Table 7:** The composition of field mud with additives.

Formulation (g)	Mixing Time, min	Base Field Drilling Mud	Modified Field Drilling Mud
Water (mL)	-	350 mL	350 mL
Bentonite	20	10 g	10 g
Sodium hydroxide	5	0.2 g	0.2 g
XC polymer	20	0.5 g	0.5 g
Starch	10	0.4 g	0.4 g
Barite	10	50 g	50 g
ArG	10	-	3.5 g

**Table 8:** Rheological properties of drilling mud.

Property	Description	Units
Plastic viscosity (PV)	θ_600_–θ_300_	cP
Initial gel (IG)	θ_3_ after 10 s	lb/100 ft^2^
Final gel (FG)	θ_3_ after 10 min	lb/100 ft^2^
Yield point (YP)	θ_300_–PV	lb/100 ft^2^

## Data Availability

Data will be made available on request.
